# Large amplicon droplet digital PCR for DNA‐based monitoring of pediatric chronic myeloid leukaemia

**DOI:** 10.1111/jcmm.14321

**Published:** 2019-06-14

**Authors:** Manuela Krumbholz, Katharina Goerlitz, Christian Albert, Jennifer Lawlor, Meinolf Suttorp, Markus Metzler

**Affiliations:** ^1^ Department of Pediatrics University Hospital Erlangen Erlangen Germany; ^2^ Department of Biology, Division of Genetics University of Erlangen‐Nuremberg Erlangen Germany; ^3^ Medical Faculty, Pediatric Hemato‐Oncology Technical University Dresden Germany

**Keywords:** *BCR‐ABL1* fusion, biomarkers, chronic myeloid leukaemia CML, digital PCR, disease monitoring, genomic fusion sequences, pediatric oncology, translocation

## Abstract

Quantification of tumour‐specific molecular markers at the RNA and DNA level for treatment response monitoring is crucial for risk‐adapted stratification and guidance of individualized therapy in leukaemia and other malignancies. Most pediatric leukaemias and solid tumours of mesenchymal origin are characterized by a relatively low mutation burden at the single nucleotide level and the presence of recurrent chromosomal translocations. The genomic fusion sites resulting from translocations are stable molecular tumour markers; however, repeat‐rich DNA sequences flanking intronic breakpoints limit the design of high sensitivity PCR assays for minimal residual disease (MRD) monitoring. Here, we quantitatively evaluated the impact of repeat elements on assay selection and the feasibility of using extended amplicons (≤1330 bp) amplified by droplet digital PCR to monitor pediatric chronic myeloid leukaemia (CML). Molecular characterization of 178 genomic *BCR‐ABL1* fusion sites showed that 64% were located within sequence repeat elements, impeding optimal primer/probe design. Comparative quantification of DNA and RNA *BCR‐ABL1* copy numbers in 687 specimens from 55 pediatric patients revealed that their levels were highly correlated. The combination of droplet digital PCR, double quenched probes and extended amplicons represents a valuable tool for sensitive MRD assessment in CML and may be adapted to other translocation‐positive tumours.

## 1 INTRODUCTION

Identification and quantification of tumour associated molecular markers is important in diagnostics and treatment response monitoring of malignant diseases. Highly sensitive assays are established and commercially available for quantification of recurrent hotspot mutations and fusion transcripts in common cancer types.

The use of genomic fusion sequences for monitoring of minimal residual disease (MRD) is laborious, because every patient needs an individually optimized quantification assay, due to the unique fusion site in each case. Furthermore, intronic repeat‐rich DNA sequences surrounding translocation breakpoints limit the design of specific and highly sensitive PCR assays in a substantial number of cases. Positioning of PCR primers outside of breakpoint flanking sequences contravenes general recommendations that short amplicons should be targeted in real‐time quantitative PCR, to ensure highly efficient amplification. To overcome these limitations, we evaluated large amplicon droplet digital PCR (laddPCR), using breakpoint spanning primers, in combination with double quenched probes, for quantification of genomic *BCR‐ABL1* fusion sequences in pediatric patients with chronic myeloid leukaemia (CML).

Ultra‐sensitive MRD assessment has become of increasing interest for patients with CML, since it was demonstrated that tyrosine kinase inhibitor treatment can be stopped indefinitely for some patients who achieve sustained deep molecular remission (DMR) for a prolonged period; however, identification of patients with the highest likelihood of continuous treatment‐free remission remains challenging. Approximately half of all individuals with CML develop disease relapse shortly after discontinuation of tyrosine kinase inhibitor (TKI) treatment,^1^, ^2^ indicating that a substantial number of quiescent CML cells remain at the time of treatment cessation, subsequently giving rise to the relapse. In addition to the well‐established methods and certified commercial techniques available for high sensitivity monitoring of *BCR‐ABL1* transcripts,^5^, ^6^ application of DNA‐based monitoring may be a useful adjuvant tool to aid treatment decisions^8^, ^9^; however, the breakpoint cluster region in the *ABL1* gene locus on chromosome 9 is particularly rich in repeat elements, and genomic breakpoints in pediatric patients with CML are (in contrast with those of adult CML patients) over‐represented within Alu repeats in the *BCR* breakpoint cluster region on chromosome 22.^12^, ^13^ These features represent challenges for the establishment of quantification assays with high specificity and sensitivity for these patients.

Here, we analysed the distribution of genomic breakpoints within repeat regions in a cohort of 178 pediatric and adolescent patients with CML. To enable DNA‐based MRD monitoring for patients with genomic fusion sites within repeat‐rich DNA sequences, we examined the benefit of laddPCR in combination with double quenched probes to overcome the technical limitations associated with conventional quantitative PCR. Comparison of fusion gene quantification by droplet digital PCR (ddPCR) in 687 blood and bone marrow samples from 55 patients with pediatric CML with results from monitoring *BCR‐ABL1* transcripts by routine quantitative reverse transcription PCR (RT‐qPCR) demonstrated that the results of both approaches were highly correlated and had high sensitivity for assessment of pediatric CML.

## 2 MATERIALS AND METHODS

### 2.1 Patients and materials

Cryopreserved mononuclear cells, fresh or frozen blood samples and bone marrow samples collected from patients with CML participating in two consecutive pediatric CML trials (CML‐paed I and CML‐paed II, EudraCT 2007‐001339‐69) were included in the analysis. Informed consent of the patients or their legal guardians was obtained, in accordance with the Declaration of Helsinki.

For identification and characterization of genomic *BCR‐ABL1* fusion sequences, 178 individuals (78 Females, 100 Males; median age: 13 years; range: 0.3‐22 years) were analysed. This cohort represents all children and adolescents diagnosed with CML in Germany during the collection period of 15 years.

### 2.2 Sequencing of genomic *BCR‐ABL1* fusion sites

DNA was isolated using the QIAamp^®^ DNA Blood Mini Kit (Qiagen, Hilden, Germany), according to the manufacturer's instructions. Genomic fusion sequences were identified using a nested multiplex long‐range PCR (MLR‐PCR) assay, as described previously.^12^

### 2.3 Analysis of breakpoint characteristics

Patient‐specific *BCR‐ABL1* fusion sequences were aligned to the human genome (hg19, UCSC Genome Browser). Breakpoint positions are listed in Table S1. Repeat elements at the fusion sites were identified using the RepeatMasker tool (http://www.repeatmasker.org/). Genomic fusion sites were then analysed for co‐localization with repeat elements, recombination‐related DNA sequence motifs, topoisomerase II binding sites, translin binding sites, heptamer/nonamer recombination signals, recombination signal sequences (Recombination Signal Sequences Site tool, http://www.itb.cnr.it/rss/), palindromic sequences (EMBOSS explorer [http://emboss.bioinformatics.nl/cgi-bin/emboss/palindrome] and palindromic sequences finder tool [http://www.biophp.org/minitools/find_palindromes/demo.php]), human minisatellite core sequences, human minisatellite conserved sequences, hypervariable minisatellite recombination sequences, DNA polymerase frameshift hotspots, immunoglobulin heavy chain class switch repeats, LTR‐IS motifs and human replication origin consensus sequences.

Components of the free software environment R (http://www.r-project.org) were used for kernel density analysis, as described previously.^14^ WebLogo Version 2.8.2. was used for graphical presentation of nucleic acid multiple sequence alignments of individual patient *BCR‐ABL1* fusion sequences, as well as patient‐specific *BCR* and *ABL1* wild‐type sequences at the corresponding positions. 15, 16.

### 2.4 Quantification of tumour‐specific genomic DNA using individual For comparative analysis of RNA‐ and DNA‐based BCR fusion sequences

For comparative analysis of RNA‐ and DNA‐based *BCR‐ABL1* therapy assessment, we evaluated a total of 687 peripheral blood or bone marrow samples from 55 individuals. Patients were selected based on the availability and quality of follow‐up samples.

Chronic myeloid leukaemia cells were analysed by detection of patient‐specific *BCR‐ABL1* fusion genes using breakpoint spanning primer and probe sets. The amplicon length of the personalized assays varied from 80 to 1330 bp. Quantification was performed by ddPCR on a QX200 reader system (BioRad) using probe‐based quantification assays with 2× ddPCR Supermix for Probes (no dUTP). A regular QX200 reaction contained 7 µL of DNA (200 ng/µL) for high sensitivity *BCR‐ABL1* detection and 1 µL of DNA (200 ng/µL) for quantification of the reference gene, albumin (*ALB*). No template control (NTC) and wild‐type negative controls were run on each plate, with adjusted amounts of DNA. All assays were conducted in duplicate. Amplitudes of FAM‐TAMRA single quenched probes were compared with those of FAM‐ZEN‐IBFQ double quenched probes, to identify the best combination for discrimination between amplicon‐negative and ‐positive droplets. A sample was regarded as positive when at least three positive droplets were detected.

For ddPCR assays with amplicon products ≥200 bp, the cycling protocol was extended to a three‐step method, with 40 cycles of 94°C for 30 seconds, 60°C for 1 minute, and 72°C for 2 minutes. To calculate the absolute number of *BCR‐ABL1* copies, fusion‐specific probe signals were normalized to that of the single copy human *ALB* gene. The sensitivities of the patient‐specific ddPCR assays for detection of *BCR‐ABL1* DNA copies were calculated based on the results from samples with the lowest quantifiable *BCR‐ABL1* copy numbers or patient‐individual dilution series, and were 0.0032%‐0.00016% (molecular response [MR], 4.5‐5.7).

### 2.5 Statistical analysis

Co‐localization of genomic breakpoints with repeat regions and DNA sequence motifs was statistically analysed using the Fisher's exact test. Differences between the ratio of mRNA% (*BCR‐ABL1* transcript/*ABL1* transcript) and DNA% (*BCR‐ABL1* fusion gene/*ALB* gene) at the day of diagnosis and 3 months after treatment initiation were assessed using the Mann‐Whitney U‐test. MRD data from quantification of the *BCR‐ABL1* fusion at the RNA and DNA levels were compared using Spearman correlation statistics.

## 3 RESULTS

### 3.1 Localization of *BCR* and *ABL1* breakpoints in repeat regions

Kernel density analyses of 178 genomic *BCR‐ABL1* fusion sequences from pediatric CML samples (breakpoint positions listed in Table S1) confirmed a bimodal distribution of genomic breakpoints within the *BCR* breakpoint cluster region. One peak of the distribution resulted from an accumulation of breakpoints in and around an Alu repeat element. Interestingly, females and males exhibited different patterns when analysed separately. *ABL1* breakpoints were evenly distributed within the breakpoint cluster region (Figure S1).

**Figure 1:**
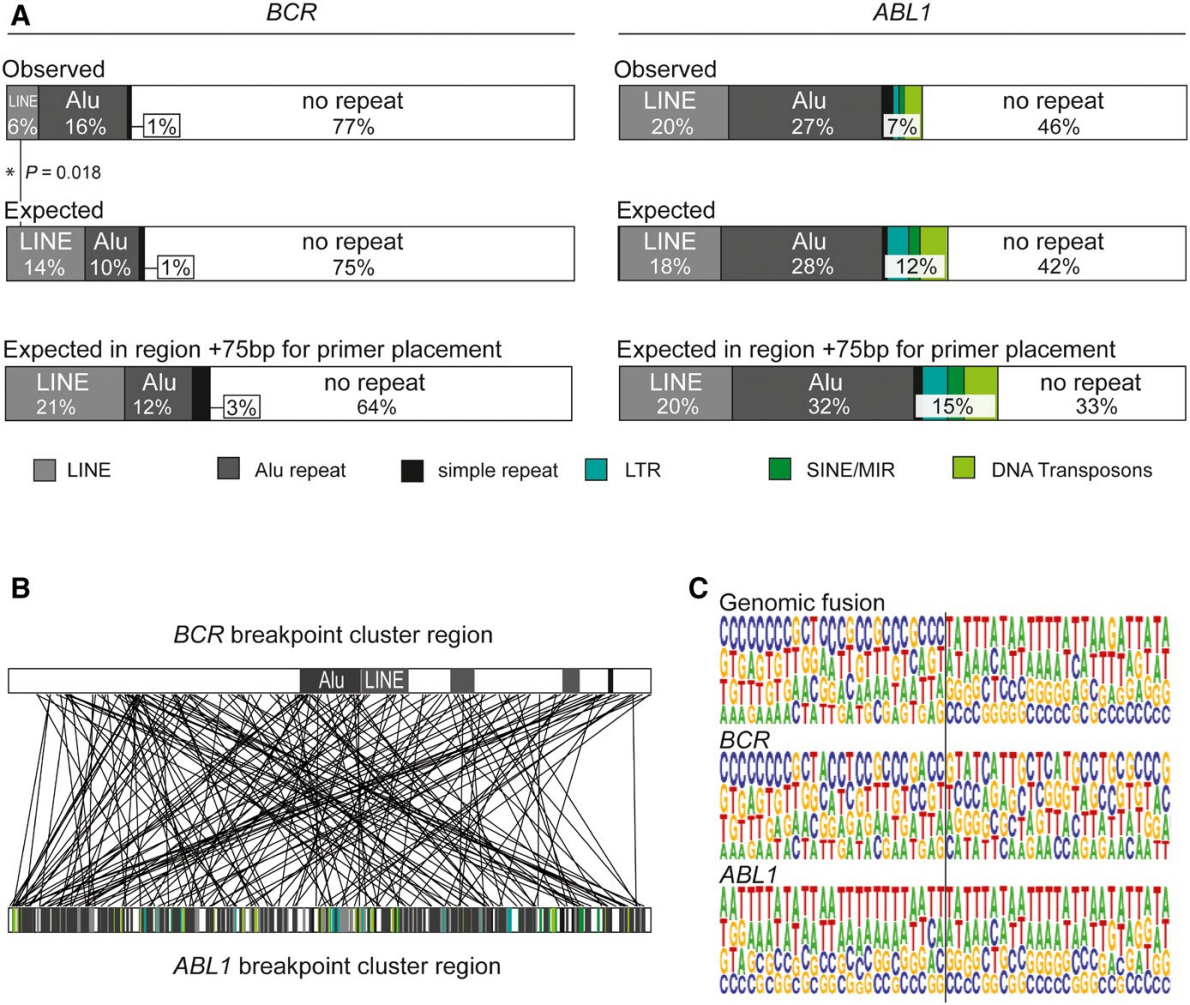
*BCR* and *ABL1* breakpoint distribution in 178 patients with pediatric CML. A, Proportion of genomic breakpoints within repeat regions in the *BCR* and *ABL1* breakpoint cluster regions, as observed in the study cohort, and expected based on the repeat content in the respective breakpoint cluster regions. The expected proportions of breakpoints within repeat elements were calculated for expanded regions, taking into account that approximately 150 bp flanking the fusion site is necessary for primer and probe design. B, Localization of genomic breakpoints within the breakpoint cluster regions in the *BCR* and *ABL1* genes in relation to repeat elements. Lines represent individual genomic breakpoints. Repeat elements are colour coded as indicated. C, Multiple alignment of *BCR‐ABL1* fusion sequences in comparison with corresponding *BCR* and *ABL1* wild‐type sequences. The black line indicates the fusion site between *BCR* (left) and *ABL1* (right)

Statistical analyses revealed no significant over‐representation of breakpoints within repeat regions or other recombination‐related sequence motifs (data not shown). Nevertheless, due to the high proportion of repeat elements in both genes involved, particularly *ABL1*, there was a high likelihood of localization of fusion sites in repeat regions. Detailed analyses showed that 23% of *BCR* breakpoints and 54% of *ABL1* breakpoints were located within these elements, mainly Alu repeats or long interspersed nuclear elements (LINEs), in accordance with the expected distribution, calculated from the overall proportion of these elements in the breakpoint cluster regions (Figure 1A). As fusion sequences are composed of both partner genes, 52% of patients with pediatric CML have fusion sites within repeat elements of one involved gene and 12% have fusion sequences located in repeat regions of both genes (Figure 1B). Multiple fusion sequence alignments of the investigated 178 fusion sites illustrated the preferred localization of breakpoints within repetitive sequences, particularly in the *ABL1* gene (Figure 1C). Only 36% of fusion sequences in pediatric CML fusion sites were not associated with repeat regions and were therefore perfectly suited for a high sensitivity, DNA‐based conventional qPCR assay design.

Extension of the target sequence to 75 nucleotides flanking either side of the DNA breakpoint (the standard sequence range required to place sense and antisense primers, and probes) reduced the proportion of fusion sequences unaffected by complicating repeat elements to 33% in the *ABL1* gene and 64% in the *BCR* gene (Figure 1A).

### 3.2 *BCR‐ABL1* fusion sequence quantification by droplet digital PCR

Highly specific and sensitive DNA quantification assays require primer and probe positions outside of repeat regions; therefore, we tested the effects on signal stability and sensitivity of increasing amplicons to lengths exceeding those conventionally used for qPCR (≥150 bp) in our probe‐based ddPCR assays.

**Figure 2:**
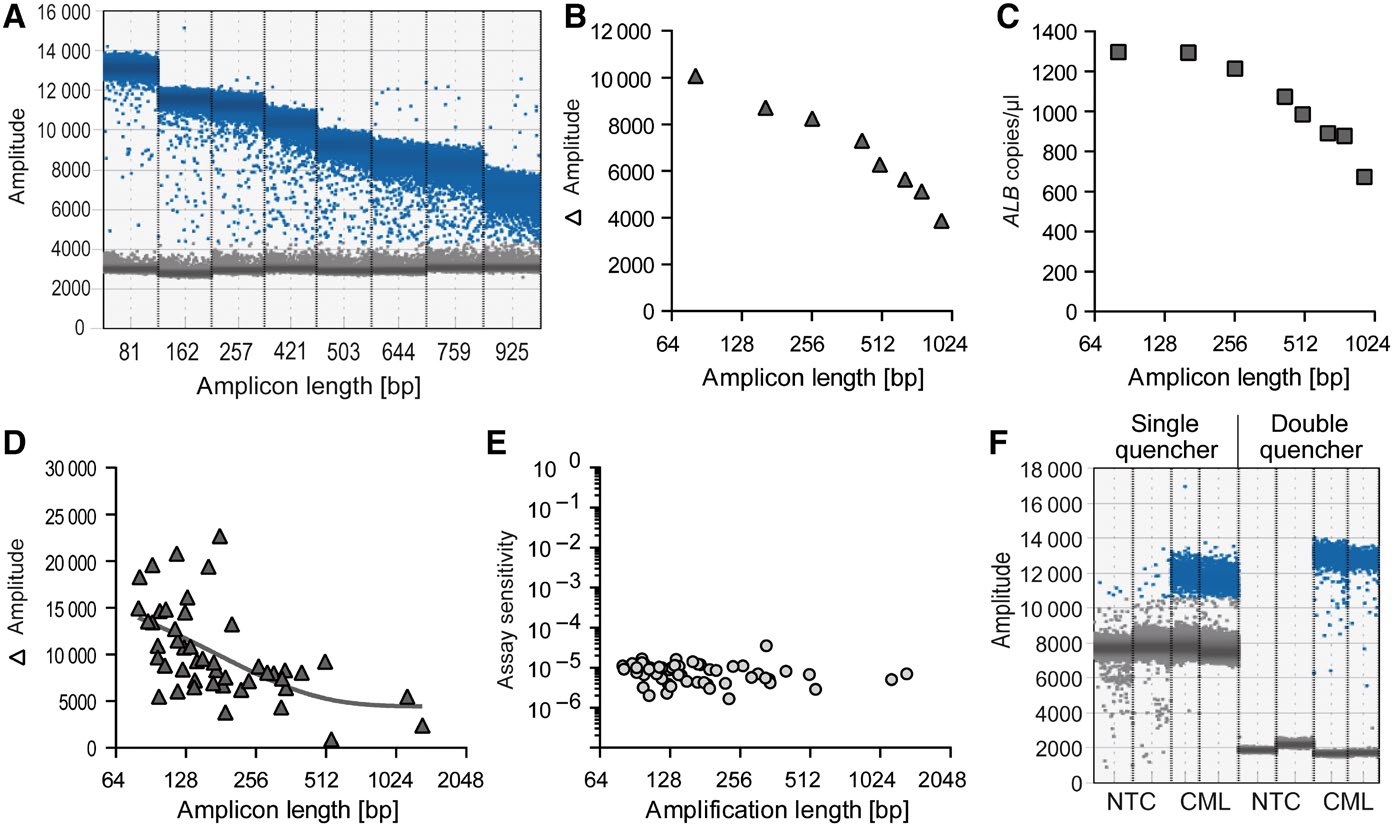
Performance of primer and probe sets with different amplicon lengths in ddPCR. A‐C, ddPCR sets with different amplicon lengths were compared for quantification of the single copy gene, *ALB*. All ddPCR sets used the same forward primer and probe, but different reverse primers, at increasing distances from the breakpoint. A, Signal amplitudes of amplicon‐positive and ‐negative droplets. B, Differences between amplitudes of amplicon‐positive and ‐negative droplets. C, Calculated concentrations (*ALB* copies/µL) of different ddPCR sets using the same amount of initial template DNA (50 ng). D and E, Comparison of amplitudes from ddPCR sets with varying amplicon lengths (D) and assay sensitivities (*BCR‐ABL1*‐positive cells/all cells) (E) for *BCR‐ABL1* fusion gene quantification in samples from patients with CML. F, Amplitudes of amplicon‐positive and ‐negative droplets using identical ddPCR sets with differently quenched probes. NTC, no template control; CML, CML patient sample

Various primer/probe combinations binding to the single copy *ALB* gene achieved highly specific quantification of the region of interest using amplicon lengths up to 925 bp, considerably beyond the typical amplicon size (≤150 bp) used for conventional qPCR assays. The absolute signal amplitude of amplicon‐positive droplets reduced with increasing amplicon length; however, separation of amplicon‐positive and ‐negative droplets (the relevant read‐out for ddPCR) remained definite and allowed reliable quantification of the target region. The sensitivity reduction on amplification of a very large amplicon (925 bp) relative to the smallest analysed amplicon (81 bp) was minimal, with a factor of 1.9 between them (Figure 2A–C).

In the next step, *BCR‐ABL1* fusion sequences were quantified in individual genomic DNA samples from 55 patients with pediatric CML. Primers were positioned beyond the usual amplicon length region of 150 bp in 26 (47%) cases, to avoid binding to a repeat element on either side of the breakpoint. As expected, the distance between amplicon‐positive and ‐negative droplets was reduced when very large amplicons were generated (Figure 2D). Nevertheless, the sensitivity of assays remained high, even for quantification of large amplicons (>1000 bp) (Figure 2E).

Reduced signal amplitudes of amplicon‐positive droplets were easily compensated for using double quenched probes, which enabled stringent separation between amplicon‐positive and ‐negative droplets (Figure 2F).

### 3.3 Comparative quantification of genomic *BCR‐ABL1* fusion genes and *BCR‐ABL1* fusion transcripts

To evaluate the potential clinical application of DNA‐based MRD monitoring for CML patients with genomic breakpoints within, and adjacent to, repeat regions, we compared DNA‐based quantification with standard RNA‐based monitoring. Amplicon lengths varied from 80 to 1330 bp for DNA‐based quantification by ddPCR.

**Figure 3:**
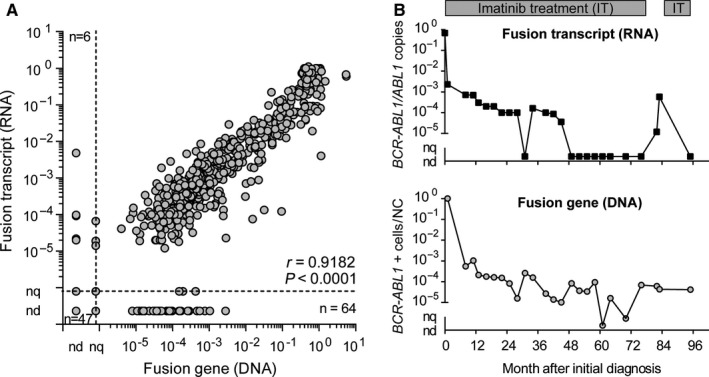
Comparison of ddPCR results from *BCR‐ABL1* fusion gene (DNA) and *BCR‐ABL1* transcript (RNA) quantification. A, *BCR‐ABL1* copy numbers in 687 blood or bone marrow samples from 55 patients with pediatric CML determined using RNA‐ and DNA‐based quantification. nc, non‐quantifiable; nd, non‐detectable. B, Example of disease monitoring by analysis of *BCR‐ABL1* fusion transcript (RNA) and *BCR‐ABL1* fusion gene (DNA) copy numbers during imatinib treatment and temporary treatment discontinuation. NC, nucleated cells

We compared 687 blood or bone marrow samples collected from 55 patients with pediatric CML at initial diagnosis and during the course of treatment. Of those 687 samples, 47 were negative at the DNA and RNA levels, while six samples were quantifiable by RT‐qPCR, but not using the DNA‐based assay. Conversely, the DNA‐based assay was able to quantify 64 samples that tested negative at the RNA level (Figure 3A). Insufficient RNA quality, due to incorrect sample handling, accounted for failed transcript quantification in 9 of 64 samples. The remaining 55 RNA‐negative samples had *BCR‐ABL1* levels around MR4.0, indicating the benefit of supplementary DNA quantification for detection of quiescent, transcript‐negative, CML cells. DNA fusion site copy number and standard RNA transcript copy number correlated well for *BCR‐ABL1* quantification (correlation coefficient, 0.9182 [*P* < 0.0001]; Figure 3A).

Figure 3B shows an exemplary course of MRD quantification for one pediatric patient, who was initially diagnosed at 6 years old. Four years after initiation of continuous TKI treatment with imatinib, *BCR‐ABL1* transcript levels decreased to non‐detectable (DMR; <MR4.5), and the patient stopped imatinib treatment 32 months later. Five weeks after treatment discontinuation, the patient lost MR4.5, indicating an imminent relapse. Retrospective quantification of the genomic *BCR‐ABL1* fusion sequence revealed persistence of the CML clone at a detectable level, illustrating the potential of supplementary DNA‐based MRD monitoring.

In addition, we investigated whether the RNA transcript/DNA fusion site ratio at diagnosis and 3 months after treatment reflected differences between good and poor responders to TKI treatment. Good responders (MR1.0, <10%, achieved 3 months after treatment initiation and MR3.0, < 0.1%, reached within 12 months of treatment initiation) exhibited lower *BCR‐ABL1* expression ratios than poor responders (Figure S2). Considering the fusion transcript type, patients with b3a2 transcripts had lower *BCR‐ABL1* expression ratios those with b2a2 transcripts at the day of initial diagnosis (*P* = 0.042), which is in contrast to results reported for adult patients.^17^ These findings require confirmation in larger cohorts.

## 4 DISCUSSION

Current technologies, for example, nested multiplex long‐range PCR assays, target enrichment next generation sequencing techniques, or MinION sequencing, allow the reliable and rapid identification of patient‐specific genomic fusion sequences,^8^, ^11^, ^12^, ^18^ facilitating the use of DNA fusion sites as highly specific molecular tumour markers for personalized MRD monitoring. In contrast to RNA‐based quantification assays, DNA‐based MRD monitoring is independent of tumour‐specific fusion gene expression and allows the quantification of absolute tumour cell numbers, as each tumour cell carries a single copy of the fusion gene; however, design of highly specific and sensitive quantification assays for MRD monitoring remains challenging when genomic fusion sites are located in repeat‐rich intronic regions, which are unfavourable for reliable target quantification.

In pediatric CML specifically, the proportion of individual fusion sites within repeat elements is very high. In addition to the high content of repeat elements within the *ABL1* breakpoint cluster region, the bimodal breakpoint distribution within the *BCR* breakpoint cluster region results in a high co‐localization of breakpoints with Alu repeats.^12^, ^13^ For specific quantification of these individual genomic fusion sequences, primers must be positioned outside of the repeat elements, resulting in amplicon lengths of several hundred nucleotides, which are incompatible with conventional real‐time PCR assays.

Reliable MRD monitoring of ultra‐low *BCR‐ABL1* levels is of increasing interest for patients with CML to identify individuals exhibiting deep molecular response who have optimal prospects for continuous treatment‐free remission. The combination of RNA‐ and DNA‐based MRD monitoring provides valuable information about transcriptional activity under inhibitor treatment and enables the quantification of transcriptionally quiescent CML cells, which are likely responsible for rapid loss of treatment‐free remission in the majority of patients with CML. 8, 9, 19, 20 Furthermore, this combined approach allows the study of *BCR‐ABL1* expression differences during the course of treatment, which may help to predict therapy outcome.^17^

Digital PCR is an end‐point PCR that enables absolute quantification of nucleic acids by Poisson statistical analysis of amplicon‐positive and ‐negative droplets, without the need for standard curves.^22^ Compared with well‐standardized real‐time PCR assays, ddPCR has equivalent sensitivity but exhibits higher robustness to PCR variations influencing amplification efficiency. This feature results in improved reproducibility and increased sensitivity, particularly when primers/probes have to be designed to target sequences with unfavourable composition.^23^

In this study, we optimized laddPCR for highly specific and sensitive assays generating amplicons up to 1330 bp, to enable the quantification of *BCR‐ABL1* fusion sequences in patients with fusion sites within or adjacent to repeat‐rich DNA stretches.

To date, laddPCR has been tested using EvaGreen dye, with moderate results. As the EvaGreen signal is proportional to the amount of double‐stranded DNA, an increase in the fluorescent signal with increasing amplicon length is expected, and could be observed in amplicons up to 300 bp. Amplicons longer than 300 bp showed a significant decrease in amplitude, with an accompanying loss of sensitivity, indicating that long products may be incompletely amplified.^24^, ^25^ Probe‐based assays for laddPCR should not be affected by this problem. Laurie *et al* tested amplicons up to 860 bp, with reliable amplitude levels for digital quantification of sequencing libraries, and observed a substantial reduction in positive droplets and hence decreasing sensitivity.^26^ In this study, we optimized laddPCR assays using double quenched probes. Using this approach, positive and negative droplets were much better separated, and quantification was feasible for large amplicons (>500 bp) with minimal reduction in sensitivity, comparable to routine RNA‐based PCR assays. To further improve the sensitivity of our ddPCR, the number of investigated wells per sample could be increased from duplicate to quadruplicate or even more parallel reactions.^7^

In summary, the availability of personalized DNA‐based therapy monitoring could be increased by quantification of genomic *BCR‐ABL1* fusion sequences using improved laddPCR with breakpoint spanning primers and double quenched probes. The sensitivity of this approach is comparable to that of established RNA‐based qPCR. Furthermore, laddPCR could be applied for therapy assessment of other chromosomal rearrangement‐positive leukaemic diseases, or in solid cancers, for detection of circulating tumour cells.

## CONFLICT OF INTEREST

The authors confirm that there is no conflict of interest.

## AUTHOR CONTRIBUTIONS

MK, MS and MM conceptualized and designed this study. MK, KG, CA and JL performed experiments. MK, MS and MM collected and analysed data. MK and MM wrote the manuscript. All authors were involved in reviewing the final version.
