# Supplementary figures and images for: Large amplicon droplet digital PCR for DNA‐based monitoring of pediatric chronic myeloid leukaemia
Source: J Cell Mol Med. 2019 Jun 14;23(8):4955–61. doi: 10.1111/jcmm.14321 (PMC6653534; doi:10.1111/jcmm.14321)

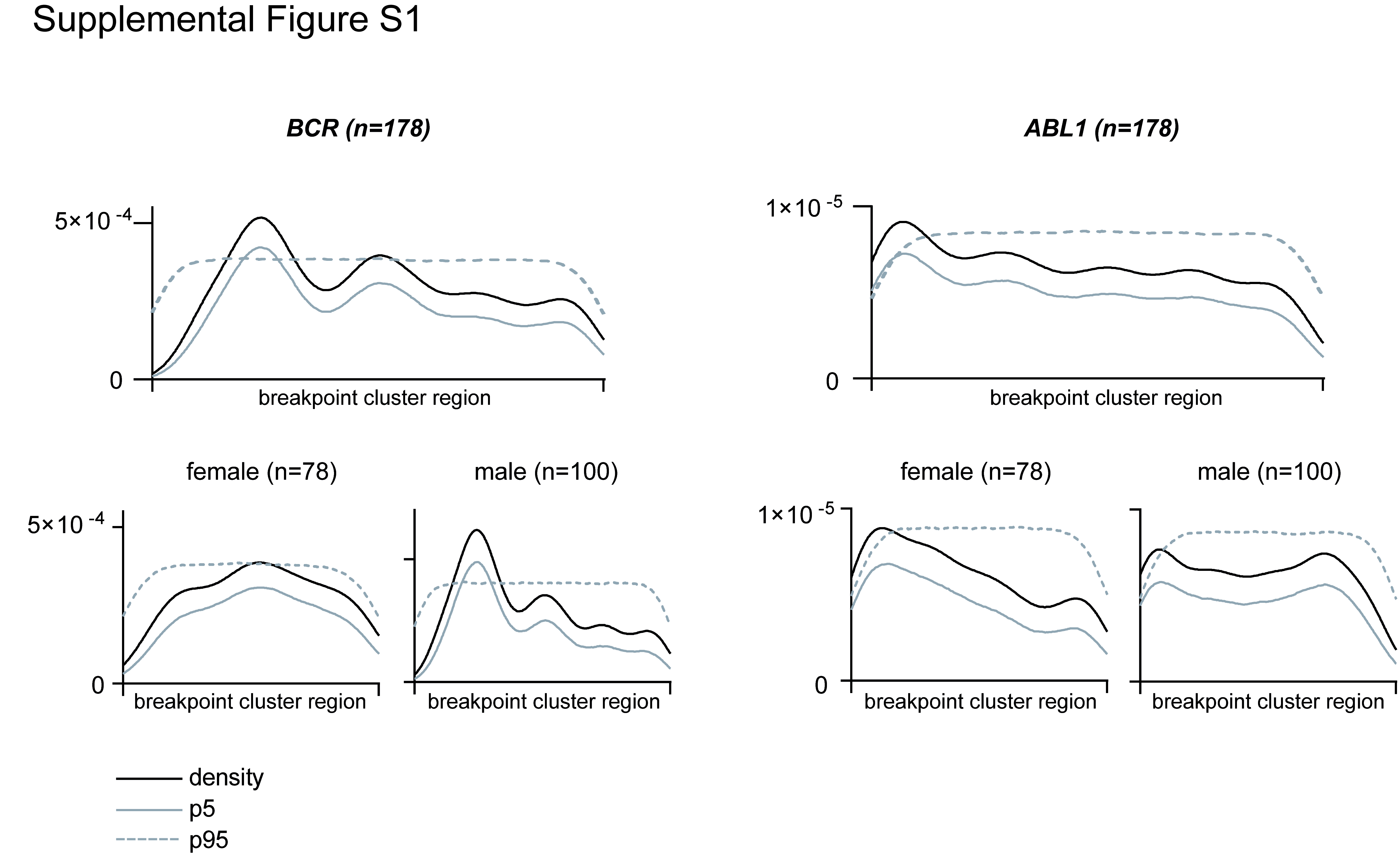

Supplement: Supplementary file 1 [file JCMM-23-4955-s001.tif]

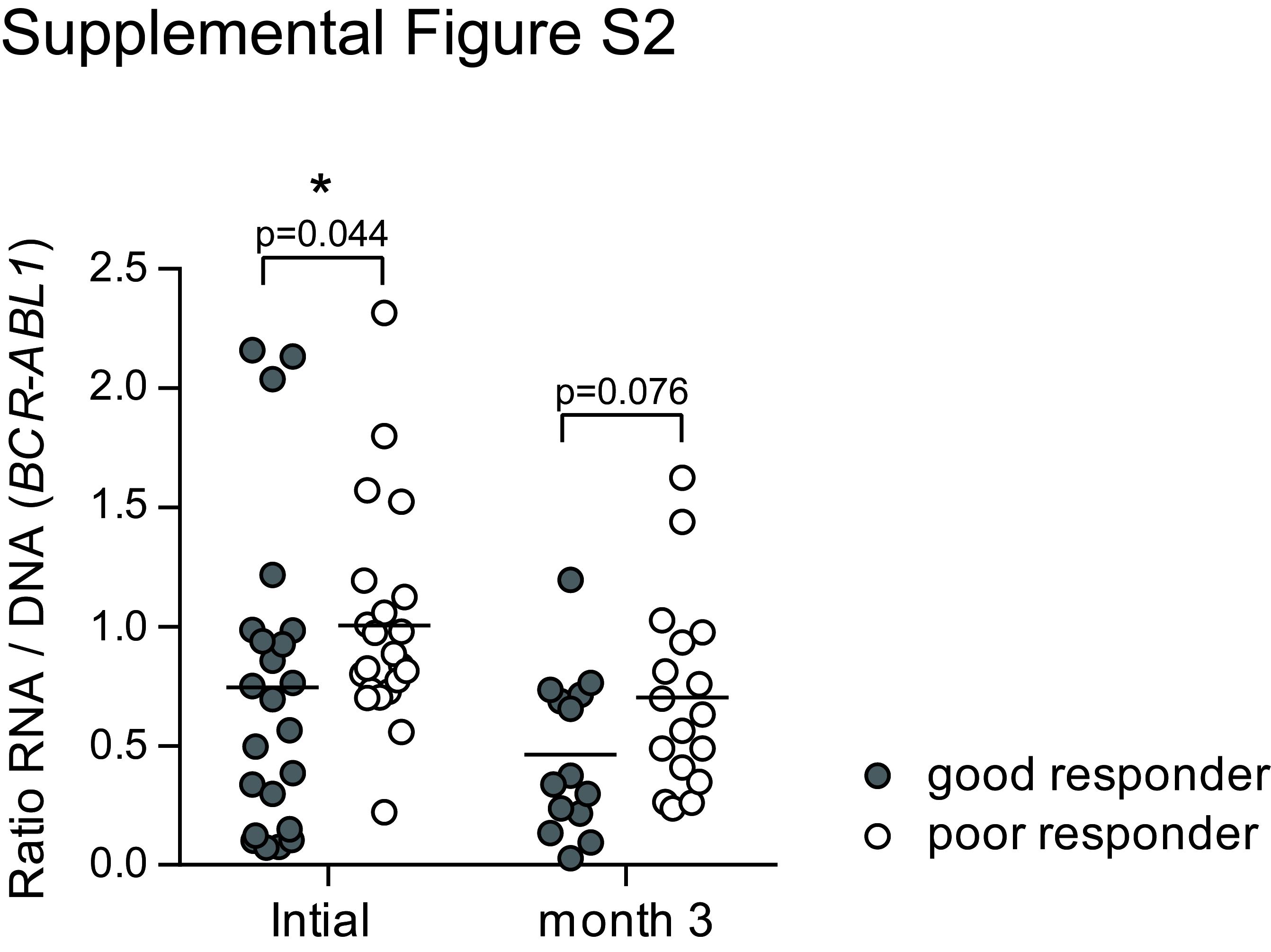

Supplement: Supplementary file 2 [file JCMM-23-4955-s002.tif]
