# Supplementary material for: Large amplicon droplet digital PCR for DNA‐based monitoring of pediatric chronic myeloid leukaemia
Source: J Cell Mol Med. 2019 Jun 14;23(8):4955–61. doi: 10.1111/jcmm.14321 (PMC6653534; doi:10.1111/jcmm.14321)
Supplement: Supplementary file 3 [file JCMM-23-4955-s003.docx]

**Supplemental Table S1:** Patient´s characteristics and genomic breakpoint positions

|  |  |  | **Genomic breakpoint position**  **(UCSC gene bank GRCh37/hg19)** | |
| --- | --- | --- | --- | --- |
| **UPN** | **Age at diagnosis [years]** | **Sex** | ***BCR* Chr22:** | ***ABL1* Chr9:** |
| 1 | 15.71 | m | 23 631 838 | 133 687 494 |
| 2 | 4.64 | m | 23 631 844 | 133 621 357 |
| 3 | 16.8 | f | 23 631 888 | 133 639 311 |
| 4 | 8.9 | f | 23 631 906 | 133 592 994 |
| 5 | 22.61 | m | 23 631 907 | 133 590 418 |
| 6 | 16.51 | m | 23 631 935 | 133 713 983 |
| 7 | 16.19 | m | 23 631 957 | 133 606 317 |
| 8 | 14.51 | m | 23 631 977 | 133 646 922 |
| 9 | 3.6 | f | 23 631 982 | 133 716 917 |
| 10 | 3.79 | m | 23 631 988 | 133 623 547 |
| 11 | 10.04 | f | 23 631 990 | 133 596 158 |
| 12 | 10.57 | f | 23 631 991 | 133 720 424 |
| 13 | 16.66 | m | 23 631 993 | 133 638 995 |
| 14 | 10.56 | m | 23 632 000 | 133 693 845 |
| 15 | 11.33 | m | 23 632 009 | 133 608 301 |
| 16 | 8.44 | f | 23 632 112 | 133 606 853 |
| 17 | 0.26 | m | 23 632 126 | 133 602 896 |
| 18 | 8.12 | f | 23 632 135 | 133 676 288 |
| 19 | 10.29 | m | 23 632 157 | 133 637 395 |
| 20 | 9.48 | m | 23 632 174 | 133 600 758 |
| 21 | 17.19 | f | 23 632 184 | 133 615 090 |
| 22 | 4.52 | m | 23 632 216 | 133 681 808 |
| 23 | 15.99 | f | 23 632 240 | 133 632 311 |
| 24 | 17.19 | m | 23 632 241 | 133 590 850 |
| 25 | 2.72 | m | 23 632 244 | 133 670 788 |
| 26 | 7.83 | m | 23 632 265 | 133 724 933 |
| 27 | 15.72 | m | 23 632 269 | 133 628 267 |
| 28 | 8.98 | m | 23 632 276 | 133 644 160 |
| 29 | 9.81 | m | 23 632 281 | 133 670 586 |
| 30 | 3.66 | m | 23 632 282 | 133 684 576 |
| 31 | 15.23 | m | 23 632 282 | 133 724 057 |
| 32 | 16.56 | f | 23 632 302 | 133 631 709 |
| 33 | 16.42 | f | 23 632 307 | 133 618 238 |
| 34 | 16.71 | m | 23 632 324 | 133 590 093 |
| 35 | 15.47 | m | 23 632 324 | 133 606 442 |
| 36 | 8.81 | m | 23 632 325 | 133 693 975 |
| 37 | 15.54 | f | 23 632 326 | 133 643 490 |
| 38 | 15.41 | m | 23 632 326 | 133 614 603 |
| 39 | 13.19 | f | 23 632 328 | 133 590 541 |
| 40 | 15.76 | m | 23 632 331 | 133 636 700 |
| 41 | 14.43 | f | 23 632 340 | 133 592 324 |
| 42 | 14.58 | m | 23 632 354 | 133 600 271 |
| 43 | 13.23 | m | 23 632 357 | 133 629 915 |
| 44 | 9.61 | f | 23 632 357 | 133 728 490 |
| 45 | 7.04 | f | 23 632 361 | 133 589 620 |
| 46 | 17.77 | m | 23 632 367 | 133 715 288 |
| 47 | 1.52 | m | 23 632 372 | 133 677 251 |
| 48 | 10.35 | f | 23 632 405 | 133 724 370 |
| 49 | 14.62 | m | 23 632 437 | 133 722 300 |
| 50 | 1.37 | m | 23 632 453 | 133 624 369 |
| 51 | 17.24 | f | 23 632 461 | 133 603 184 |
| 52 | 14.3 | m | 23 632 497 | 133 647 115 |
| 53 | 17.93 | m | 23 632 504 | 133 696 779 |
| 54 | 14.53 | m | 23 632 507 | 133 646 084 |
| 55 | 7.56 | m | 23 632 511 | 133 594 048 |
| 56 | 15.71 | f | 23 632 512 | 133 684 699 |
| 57 | 10.47 | m | 23 632 517 | 133 642 668 |
| 58 | 0.86 | m | 23 632 557 | 133 590 997 |
| 59 | 15.76 | m | 23 632 558 | 133 689 848 |
| 60 | 15.28 | f | 23 632 564 | 133 614 220 |
| 61 | 7.69 | m | 23 632 575 | 133 689 957 |
| 62 | 8.3 | m | 23 632 579 | 133 659 793 |
| 63 | 9.69 | m | 23 632 587 | 133 593 672 |
| 64 | 7.26 | f | 23 632 589 | 133 714 129 |
| 65 | 15.56 | m | 23 632 601 | 133 720 340 |
| 66 | 22.67 | m | 23 632 669 | 133 591 109 |
| 67 | 15.11 | m | 23 632 721 | 133 686 163 |
| 68 | 14.44 | m | 23 632 736 | 133 628 969 |
| 69 | 7.76 | f | 23 632 768 | 133 676 171 |
| 70 | 7.44 | f | 23 632 785 | 133 726 841 |
| 71 | 8.17 | f | 23 632 812 | 133 704 199 |
| 72 | 4.99 | m | 23 632 823 | 133 591 048 |
| 73 | 16.28 | f | 23 632 828 | 133 681 958 |
| 74 | 10.3 | m | 23 632 840 | 133 658 779 |
| 75 | 15.4 | m | 23 632 870 | 133 631 439 |
| 76 | 10.49 | m | 23 632 877 | 133 669 375 |
| 77 | 4.09 | f | 23 632 929 | 133 620 423 |
| 78 | 13.26 | f | 23 632 970 | 133 693 375 |
| 79 | 16.67 | m | 23 632 996 | 133 713 859 |
| 80 | 14.52 | f | 23 633 006 | 133 660 327 |
| 81 | 15.27 | f | 23 633 035 | 133 606 710 |
| 82 | 14.17 | f | 23 633 053 | 133 663 499 |
| 83 | 14.28 | m | 23 633 091 | 133 659 939 |
| 84 | 10.05 | m | 23 633 098 | 133 700 610 |
| 85 | 3.33 | f | 23 633 114 | 133 694 468 |
| 86 | 18.6 | f | 23 633 115 | 133 599 915 |
| 87 | 6.77 | f | 23 633 124 | 133 596 860 |
| 88 | 16.58 | m | 23 633 125 | 133 694 482 |
| 89 | 14.25 | m | 23 633 131 | 133 629 625 |
| 90 | 14.31 | f | 23 633 148 | 133 590 210 |
| 91 | 16.59 | m | 23 633 152 | 133 599 905 |
| 92 | 14.96 | m | 23 633 155 | 133 625 331 |
| 93 | 12.92 | m | 23 633 159 | 133 625 331 |
| 94 | 3.98 | m | 23 633 162 | 133 711 235 |
| 95 | 12.35 | m | 23 633 163 | 133 694 511 |
| 96 | 14.63 | f | 23 633 167 | 133 648 487 |
| 97 | 13.39 | m | 23 633 174 | 133 720 529 |
| 98 | 8.84 | f | 23 633 202 | 133 656 650 |
| 99 | 18.98 | f | 23 633 219 | 133 633 827 |
| 100 | 14.8 | m | 23 633 246 | 133 670 251 |
| 101 | 6.28 | f | 23 633 292 | 133 674 686 |
| 102 | 12.88 | f | 23 633 319 | 133 596 464 |
| 103 | 13.4 | f | 23 633 324 | 133 704 782 |
| 104 | 5.44 | f | 23 633 332 | 133 637 034 |
| 105 | 12.23 | m | 23 633 353 | 133 701 860 |
| 106 | 1.23 | f | 23 633 365 | 133 638 627 |
| 107 | 18.22 | m | 23 633 376 | 133 667 600 |
| 108 | 13.5 | m | 23 633 377 | 133 593 359 |
| 109 | 4.5 | m | 23 633 385 | 133 695 482 |
| 110 | 7.76 | f | 23 633 398 | 133 652 418 |
| 111 | 11.95 | f | 23 633 398 | 133 666 186 |
| 112 | 15.2 | m | 23 633 410 | 133 609 600 |
| 113 | 17.62 | m | 23 633 426 | 133 651 603 |
| 114 | 1.86 | m | 23 633 440 | 133 626 365 |
| 115 | 9.22 | m | 23 633 454 | 133 619 938 |
| 116 | 11.34 | m | 23 633 535 | 133 704 474 |
| 117 | 9.02 | f | 23 633 539 | 133 662 417 |
| 118 | 9.45 | m | 23 633 557 | 133 591 119 |
| 119 | 15.23 | f | 23 633 569 | 133 631 740 |
| 120 | 13.09 | m | 23 633 580 | 133 657 152 |
| 121 | 15.66 | f | 23 633 581 | 133 614 775 |
| 122 | 8.74 | f | 23 633 603 | 133 723 393 |
| 123 | 11.03 | f | 23 633 613 | 133 669 914 |
| 124 | 9.33 | f | 23 633 644 | 133 606 071 |
| 125 | 15.15 | m | 23 633 710 | 133 674 522 |
| 126 | 13.16 | f | 23 633 724 | 133 665 018 |
| 127 | 12.13 | f | 23 633 748 | 133 597 924 |
| 128 | 14.23 | m | 23 633 784 | 133 615 938 |
| 129 | 4.72 | f | 23 633 804 | 133 647 320 |
| 130 | 15.92 | f | 23 633 830 | 133 650 031 |
| 131 | 11.92 | m | 23 633 831 | 133 697 448 |
| 132 | 12.55 | m | 23 633 860 | 133 709 898 |
| 133 | 10.02 | m | 23 633 878 | 133 714 487 |
| 134 | 7.59 | f | 23 633 884 | 133 651 897 |
| 135 | 8.67 | f | 23 633 896 | 133 727 563 |
| 136 | 17.56 | f | 23 633 906 | 133 688 088 |
| 137 | 15.09 | m | 23 633 937 | 133 693 486 |
| 138 | 7.34 | f | 23 633 952 | 133 719 160 |
| 139 | 17.33 | m | 23 633 979 | 133 593 100 |
| 140 | 14.82 | f | 23 633 981 | 133 611 069 |
| 141 | 12.19 | m | 23 633 997 | 133 688 193 |
| 142 | 7.34 | m | 23 634 012 | 133 661 064 |
| 143 | 13.66 | f | 23 634 064 | 133 604 656 |
| 144 | 11.29 | m | 23 634 079 | 133 713 155 |
| 145 | 9.82 | m | 23 634 103 | 133 727 053 |
| 146 | 17.12 | m | 23 634 132 | 133 669 903 |
| 147 | 15.27 | f | 23 634 168 | 133 711 590 |
| 148 | 13.69 | f | 23 634 171 | 133 628 886 |
| 149 | 17.25 | f | 23 634 173 | 133 601 807 |
| 150 | 15 | m | 23 634 207 | 133 603 704 |
| 151 | 14.52 | f | 23 634 227 | 133 684 784 |
| 152 | 14.95 | f | 23 634 232 | 133 642 322 |
| 153 | 16.97 | m | 23 634 275 | 133 705 195 |
| 154 | 15.68 | f | 23 634 353 | 133 633 799 |
| 155 | 9.29 | m | 23 634 356 | 133 696 890 |
| 156 | 17.61 | f | 23 634 379 | 133 596 211 |
| 157 | 13.04 | f | 23 634 403 | 133 677 312 |
| 158 | 10.13 | m | 23 634 425 | 133 671 974 |
| 159 | 14.25 | m | 23 634 445 | 133 641 296 |
| 160 | 16.02 | m | 23 634 457 | 133 606 574 |
| 161 | 15.7 | f | 23 634 515 | 133 598 349 |
| 162 | 6.37 | f | 23 634 533 | 133 618 128 |
| 163 | 6.65 | f | 23 634 549 | 133 704 274 |
| 164 | 14.73 | f | 23 634 575 | 133 607 565 |
| 165 | 7.46 | f | 23 634 586 | 133 633 318 |
| 166 | 17.74 | m | 23 634 587 | 133 601 254 |
| 167 | 10.76 | f | 23 634 587 | 133 662 301 |
| 168 | 13.9 | m | 23 634 593 | 133 599 843 |
| 169 | 17.83 | m | 23 634 622 | 133 687 600 |
| 170 | 11.35 | m | 23 634 643 | 133 591 574 |
| 171 | 8.9 | f | 23 634 662 | 133 624 148 |
| 172 | 17.32 | m | 23 634 725 | 133 660 249 |
| 173 | 14.24 | m | 23 634 726 | 133 591 620 |
| 174 | 6.53 | f | 23 634 727 | 133 726 723 |
| 175 | 16.27 | m | 23 634 742 | 133 657 587 |
| 176 | 17.72 | f | 23 634 773 | 133 637 666 |
| 177 | 10.17 | m | 23 634 808 | 133 667 088 |
| 178 | 5.04 | f | 23 634 817 | 133 622 085 |
