# Supplementary material for: Large amplicon droplet digital PCR for DNA‐based monitoring of pediatric chronic myeloid leukaemia
Source: J Cell Mol Med. 2019 Jun 14;23(8):4955–61. doi: 10.1111/jcmm.14321 (PMC6653534; doi:10.1111/jcmm.14321)
Supplement: Supplementary file 4 [file JCMM-23-4955-s004.docx]

**Supplemental Figure legends**

*Supplemental FigureS1*

Results of Kernel density analysis for the breakpoint distribution in *BCR* and *ABL1* breakpoint cluster regions (178 pediatric CML patients); dashed line = breakpoint density; gray line = lower limit of 95% confidence band determined by bootstrapping procedure; black line = 95% confidence interval of a density function resulting from simulations at randomly distributed pseudo-breakpoints.

*Supplemental FigureS2*

*BCR-ABL1* transcript / *ABL1* transcript (RNA%) and *BCR-ABL1* fusion gene / *ALB* gene (DNA%) ratio at the day of diagnosis and 3 months after treatment start. Differences between good responders (MR1.0 (<10%) achieved 3 months after treatment start and MR3.0 (<0.1%) achieved 12 months after treatment start) and poor responders.
